# Exposition of respiratory ailments from trace metals concentrations in incenses

**DOI:** 10.1038/s41598-021-89493-w

**Published:** 2021-05-21

**Authors:** A. H. Bu-Olayan, B. V. Thomas

**Affiliations:** Department of Chemistry, Kuwait University, Khaldiya Campus, BLDG 48, RM 7, PB 5969, 13060 Safat, Kuwait

**Keywords:** Environmental impact, Epidemiology, Environmental monitoring, Pollution remediation

## Abstract

Selected trace metals of importance in different incense before and after the smoldering process were assessed based on the recent respiratory ailments. Marketed perfumed and non-perfumed incense from different countries was separately analyzed using an Inductive coupled Plasma-Mass Spectroscopy (ICP-MS). A particulate analyzer measured the dispersed particulates (PM_2.5_) in the indoor environment. The analysis revealed higher mean metals concentrations in the smoldered perfumed incense (1.98 µg g^−1^) than in the non-smoldered and non-perfumed incense (0.59 µg g^−1^). Pilot-scale experiments included the smoke dispersed in different sized-rooms, and the distance between the inhaler and the smoldering incense. Simultaneously, a questionnaire distributed to 300 residents from six sampling areas revealed the exposure of incense to human health. This study indicated significant attributes on (a) the room size and incense volume, (b) the permissible inmate’s number in a room, (c) the distance between the inmate vicinity and the point of smoldering incense, (d) selectivity of incense, besides the outdoor environmental influence. Furthermore, this study revealed the various categories of respiratory ailments in residents in relation to the frequency of burning incense, prolonged smoke exposure, and the impact of burners although, earlier beneficial effects of incenses were evidenced. This study recommends preventive measures to human respiratory ailments from smoldering incense.

## Introduction

Global industrialization, urbanization, and escalation of the population over the past decade has ominously deteriorated the quality of indoor and outdoor air and has become an important health issue to humans. Advancement in digital technology enhanced people to spend their major time indoor irrespective of their working place, public, at home, or institutes and even during their spare time. Thus, exposition to indoor contaminants was discernible. Indoor air is characterized mainly from smoke through cooking and smoking cigarettes, and secondarily by incense burning. However, in an inadequately ventilated residence, it is hypothesized that the incense smoke that has heavy metals concentrations and other organic compounds may exceed the metals concentrations of cigarette smoke^1^.

The residents of many Middle East countries follow the customary practice of smoldering perfumed or non-perfumed scented wood (‘Bakhour’) or naturally available resin (Frankincense) incenses. Citizens use different types of incense for pleasant aroma, to mask bad odors, as stress relaxant or health rejuvenators^2–14^. The best aromatic oils from the resin, *Boswellia sacra,* are produced from the Salalah coastal plain and Dhofar Valley of Oman.

Several studies^15–20^ showed these incenses to (a) ease stress, (b) balance the immune system, (c) decrease inflammation and, (d) anti-aging. Recent studies^18,21–27^ revealed the deleterious effect of incense smoke on humans. The incense smoke includes gaseous pollutants (CO, NO_x_, SO_x_), organic compounds and toxic inorganic pollutants (PAHs, benzene, heavy metals). Researchers^24,28–34^ observed lung cancer because of excess particulate concentrations (PM_10_) from the incense smoke compared to the tobacco smoke. However, their studies did not evidence the indirectly inhaled incense smoke to that of the direct cigarette smoke inhalation and trace metals contamination and hence, this investigation.

The choice of incenses selection by people varies with culture, tradition, and personal sense to the aroma. Direct incense burning involves direct ignition producing flame instead of an external source that involves heat and flame. The burning ash on the incense blazes the rest of the incense. The indirect incense burning uses charcoal or electricity as the heat sources^35,36^. The texture of the material in the incense tends to change the duration of its burning period. Finer ingredients burn more rapidly than coarse materials because their total surface area is less.

**Figure 1:**
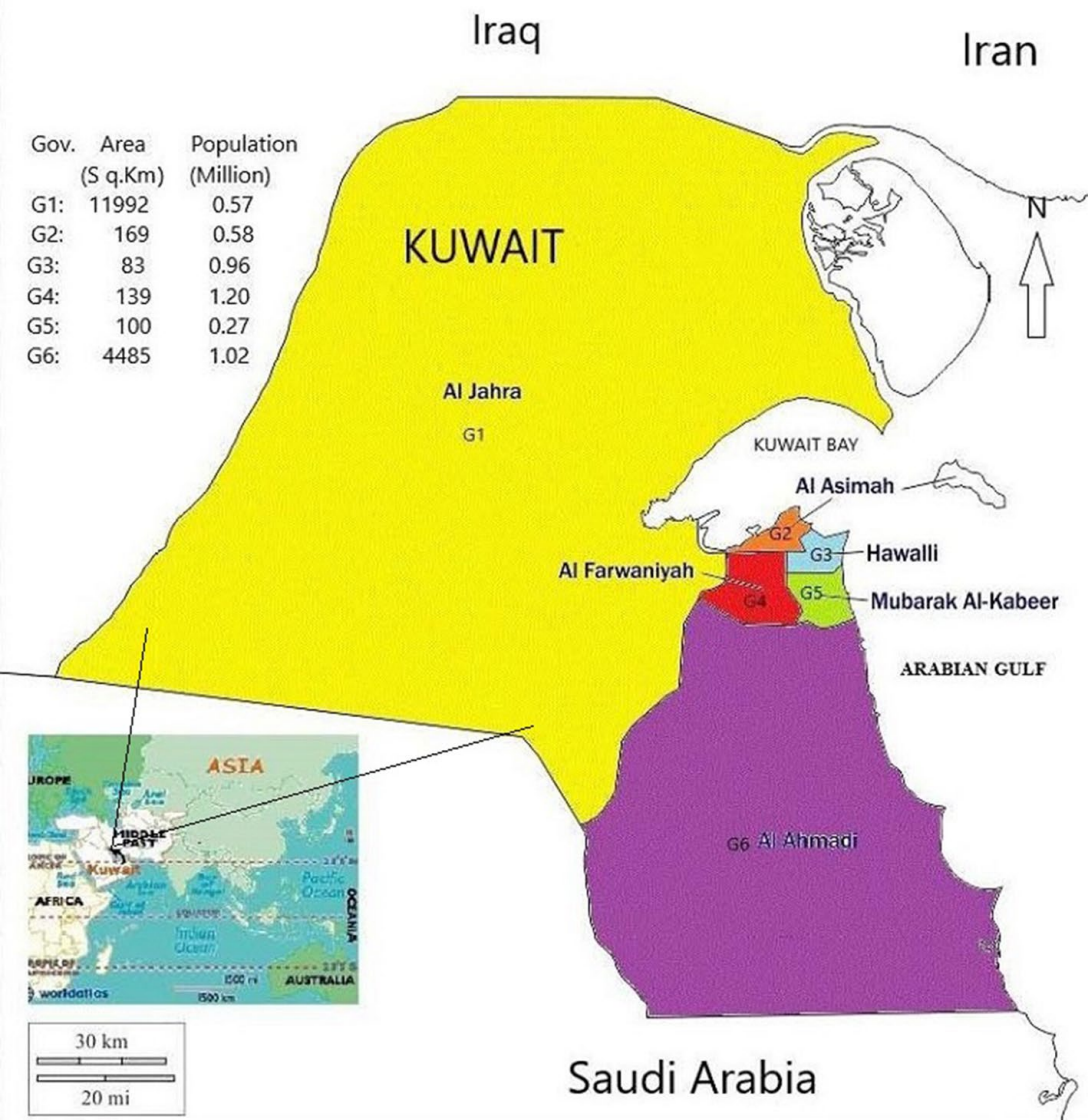
Six Kuwait Governorates selected for sampling. G1–G6: Governorates: Al-Jahra, Al-Asimah, Al-Hawalli, Farwaniya, Mubarek Al-Kabeer, Al-Ahmedi. This map available publicly is generated using MS-Paint (MS-Office-365).

Kuwait is geographically categorized by Six Governorates (Fig. 1). Observations showed an increasing trend in the practice of burning ‘Bakhour,’ Frankincense and, incense sticks. The Kuwait market imports ‘Bakhour’ and Frankincense from the State of Oman, Saudi Arabia, Yemen, Cambodia, India, and the United Arab Emirates. Although air contaminant standards were set by statutory bodies^37^, the local statutory bodies did not undertake any regulated measures to burning incenses. The effects of burning such incense in Kuwait were least described^30,31,38,39^. Following the respiratory ailments^38,42^, this study: (a) conducted a survey on the marketed incense apportioned in the six Kuwait Governorate areas, (b) analyzed selected trace metals of importance such as chromium (Cr), nickel (Ni), lead (Pb), Arsenic (As), Vanadium (V), and cadmium (Cd) in the perfumed and non-perfumed, smoldered and non-smoldered incense, (c) surveyed using a questionnaire to assess the pollution impact and contributing factors of the frequently used incense by the indoor residents and, (d) deduced the possible alleviating measures to respiratory ailments.

## Methods

### Sample location

Imported incense samples were selected from seven countries distributed in the local markets of the six Kuwait Governorate (G1–G6) areas (Fig. 1) of pollution importance [vicinity to thermal and coal plants (G1), small to large-scale industries and waste treatment plants (G2), thickly populated residential area (G3–G5), and oil sector (G6)] based on the incense availability in the market throughout the study period.

### Sample analysis by primary direct method

Incense from seven countries of origin of samples distributed in the local markets of six Kuwait Governorate areas were collected and stored separately in sterile plastic containers. They were categorized into eight non-perfume-soaked (six Frankincense resins and two Oudh wood-*Aquilaria* species) and Eight perfume soaked (‘Bakhour’) incense for trace metals analysis in the laboratory. Replicates (5 g) of each non-smoldered incense samples dried in an oven (GallenKamp-II) at 40 °C for 1 h (to remove moisture if any), were powdered and sieved in a #18 mesh of 1.0 mm size to enable the sample uniformity. A pilot-scale experiment with replicates ranging from 0.2 g to10 g) of incense were smoldered in a sterile glass chamber (0.72 ft length × 0.32 ft width × 0.72 ft height) to quantify the incense that will produce the permissible tolerable and toxic limits to safe human inhalation. The smoke was collected from the chamber using a Tedlar bag and passed through a real-time environmental particulate air monitor (EPAM-5000, Hazdust, USA) to measure the PM_2.5_ by nephelometry and, by gravimetry on an EPA-FRM-style 47 mm filter cassette. This experiment was standardized for (a) the time taken to disperse the smoke uniformly in the varying room sizes (100 ft^2^-180 ft^2^), (b) the effect of incense dispersion within 0.5 ft to 1.0 ft length from the inmate’s point of contact, (c) the effect of incense smoke in the presence of ventilation and, (d) other indoor governing parameters that were achieved by indirect method assessment. These non-smoldered, smoldered and ash of incenses were acid digested (3% w/v HNO_3_) in a microwave digester (Ethos-1, Milestone, Italy) and the digested solution was analyzed for trace metals concentrations in the ICP-MS (NexION-350, Perkin Elmer, Inc., USA). Quality assurance followed using blanks, triplicate samples, controls, and standard reference material (SRM 1547- Peach leaves, NIST, USA). Trace metals concentrations with > 90% sample recovery to that of the SRM confirmed the instrument’s precision, consistency, and reproducibility of the experiments^8,37^.

### Validation of oil in the samples by indirect method

The vendors labeled the percentage or concentrations of oil in the marketed incense as standardized by the local statutory bodies. Few low-quality incenses never indicated the oil content. Earlier data^40,41^ from such incense was validated with the present trace metals concentrations in the samples. The country-wise raw incense was purchased following the database of oil in them. They were spiked and seasoned with the appropriate concentrations and volume of oil. They were dried in natural condition for 10–15 days and trace metals concentrations determined as described in the previous section. The analysis was conducted both before and after smoldering, perfumed and non-perfumed incenses.

### Non-parametric validation of samples

The indirect method of analysis followed using a questionnaire distributed to residents of six Kuwait Governorate areas. The response to the questionnaire followed the ethical practices outlined by the local statutory bodies, and after obtaining the mutual consent of the residents. The questionnaire detailed the information on the respondent’s area-wise habitation, the size of the rooms, burning frequency of incense, burning methods, and the type of burners. The mean trace metals concentrations in the ash content of incenses smoldered using the charcoal, direct flame and electric burners was analyzed to characterize the differential effect from the three burners. Additionally, this study assessed trace metals concentrations in relation to the room size (ft^2^), Governorate-wise PM_2.5_ before, and after burning incense, and the type and intensity of respiratory ailments.

### Statistical analysis

All data were processed with MS-Excel 365. The comparative analysis between replicates of smoldered, non-smoldered, perfumed, and non-perfumed incenses was facilitated by separately analyzing the total mean trace metals concentrations. Statistical tests represented in the figures included standard error and means for each variable. The mean of each trace metal was determined to reveal the sequence of magnitude from high to low concentrations. The significant differences between the different variables such as country-wise, Governorate-wise, oil content in metals to that of the smoldered, non-smoldered, perfumed, and non-perfumed incenses were statistically validated using the ANOVA tests.

### Ethics declaration

- The study is approved by the review committee on projects, Research Administration (RA), Kuwait University on human subjects.
- A signatory informed consent from the residents of Kuwait on the use of incenses and general health concerns was obtained while maintaining personal privacy using an offline questionnaire survey (non-parametric) method.
- All study methods were carried out in accordance with relevant guidelines and regulations.

## Results and discussion

Earlier studies observed the abundant use of frankincense and ‘Bakhour’ (aromatic Oudh wood) in the Middle East countries^2–6,9,10,12,13,15–17,20,41^. Among the eight non-smoldered (before burning) non-perfumed incenses, the total mean trace metals concentrations (1.05 µg g^−1^) were high in Oudh wood procured from Somalia and low total mean concentrations (0.47 µg g^−1^) from samples of Yemen. Irrespective of the source of origin, trace metals concentrations in the Oudh wood was in the sequence of Pb > Ni > Cr > As > Cd > V. Country-wise analysis revealed high trace metals concentrations in non-smoldered non-perfumed frankincense in the sequence of the Northern Oman (0.97 µg g^−1^) > Saudi Arabia (0.72 µg g^−1^) > India (0.60 µg g^−1^) > Indonesia (0.38 µg g^−1^) > Cambodia (0.32 µg g^−1^) > Southern Dhofar-Oman (0.26 µg g^−1^). The high trace metals concentrations in incense from these countries attribute to (a) the absorption and translocation of pollutants through the roots, barks, stem, leaves, and resin of frankincense trees from the atmosphere, (b) the contamination of trace metals during the collection and storage process of the Oudh wood and frankincense resin, besides, the shelf life period and, (c) species specificity and quality of the resin that retained the trace metals concentrations. These findings agreed with the earlier studies^12,14,17,19^.

**Figure 2:**
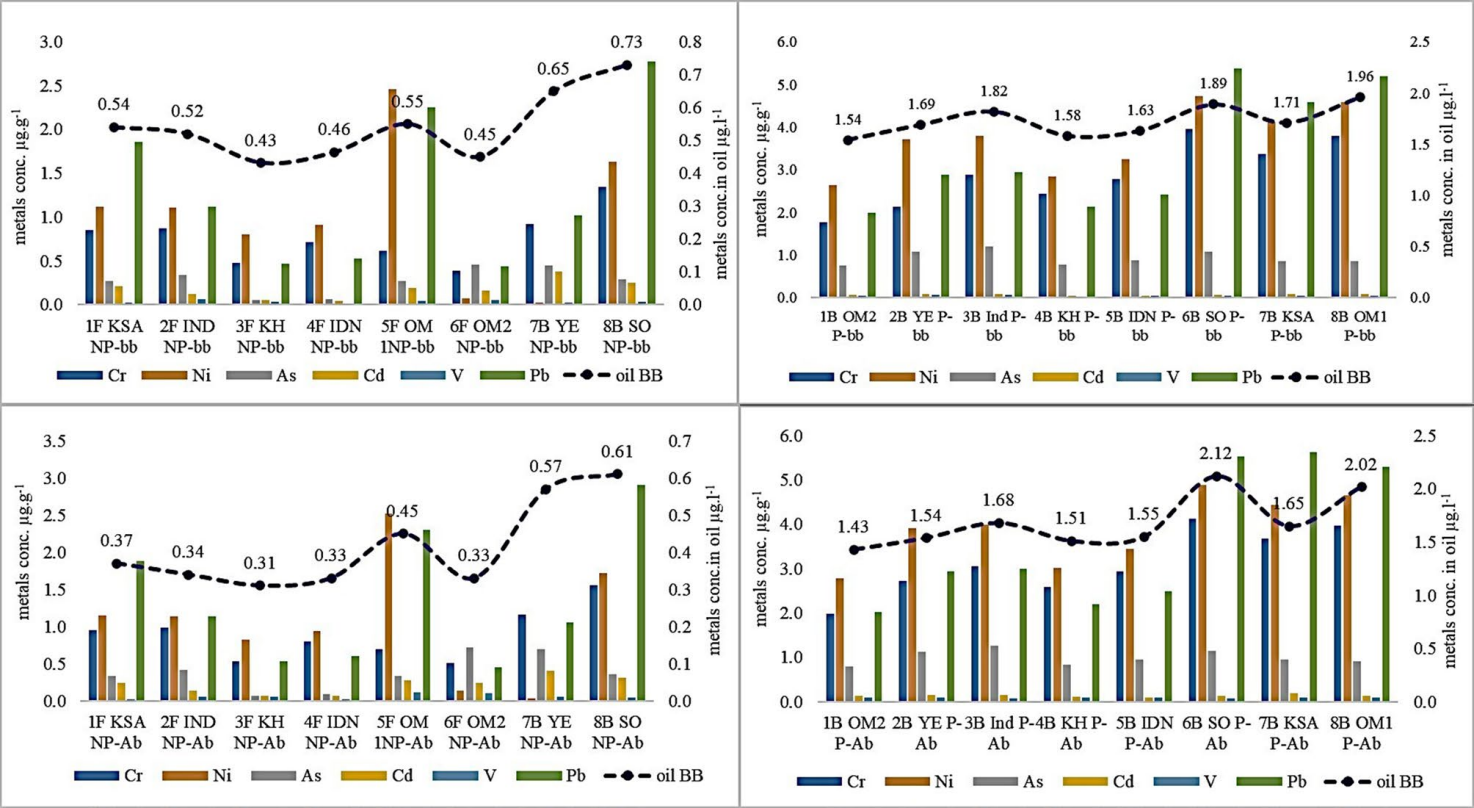
Country-wise, and metals-wise before and after smoldering in non-perfumed and perfumed incense. *P* perfumed, *NP* non-perfumed; *IDN* Indonesia, *YE* Yemen, *SO* Somalia, *IND* India, *OM1* Oman-Dhofar, *OM2* Oman-Salalah; *KH* Cambodia, *KSA* Saudi Arabia; *F* frankincense, *B* Bokhur, *bb* before smoldering; *Ab* after smoldering.

In the eight non-smoldered perfumed incense, the trace metals concentrations (2.54 µg g^−1^) were observed high in Oudh wood from Somalia compared to the low concentrations (1.21 µg g^−1^) in Oman, respectively (Fig. 2). The residents cherished the natural fragrance of the frankincense resins and hence, never perfumed. The trace metals concentrations in frankincense were lower than the metals concentrations in Oudh wood irrespective of the soaked perfumes.

### Trace metals concentrations in smoldered non-perfumed and perfumed incense

Among the eight smoldered (after burning) non-perfumed incense, trace metals concentrations were in the sequence of Ni > Pb > Cr > As > Cd > V. High trace metals concentrations (1.15 µg g^−1^) were in the Oudh wood procured from Somalia in contrast to the low metals concentrations (0.57 µg g^−1^) in Yemen, respectively. Trace metals concentrations (1.04 µg g^−1^) were found high in smoldered non-perfumed frankincense obtained from the Northern Oman and low metals concentrations (0.35 µg g^−1^) in frankincense procured from Cambodia. In the case of smoldered perfumed incense, high trace metals concentrations were observed in Oudh wood of Somalia compared to the low high trace metals concentrations in the Oudh wood of Northern Oman. In an overall view, trace metals concentrations were higher in smoldered perfumed incense than the non-smoldered non-perfumed incense. The analysis revealed three times the total mean trace metals concentrations (1.98 µg g^−1^) in the smoldered perfumed incense attributing to the influence of added contaminants than the total mean metals concentrations (0.66 µg g^−1^) in the smoldered non-perfumed incense samples. Likewise, the total mean trace metals concentrations from the non-smoldered perfumed incenses (1.85 µg g^−1^) was higher than the total mean trace metals concentrations from non-smoldered non-perfumed (0.60 µg g^−1^) thus, indicating the influence of oil in such incenses. Studies^16,30^ showed such additive pollutants in the incense attributed to the high organic constituents namely, essential oil content in the non-smoldered non-perfumed or perfumed incense compared to the smoldered incense (Fig. 2). Furthermore, analysis on the oil that was used to label the incenses from the local market revealed the high trace metals concentrations in the oil-soaked (perfumed) incense than in the incense with low oil (non-perfumed) that is described below:

- non-smoldered perfumed (1.73 µg l^−1^) Vs non-smoldered non-perfumed incense (0.54 µg l^−1^),
- non-smoldered non-perfumed (0.54 µg l^−1^) Vs smoldered non-perfumed incense (0.41 µg l^−1^),
- non-smoldered perfumed (1.73 µg l^−1^) Vs smoldered perfumed incense (1.69 µg l^−1^),
- smoldered perfumed (1.69 µg l^−1^) Vs smoldered non-perfumed incense (0.41 µg l^−1^),

The low trace metals concentrations in smoldered oil-soaked incense could be attributed to the oil evaporation. This study also statistically validated the significance of the oil spiked incenses compared to their non-oil spiked incenses by ANOVA tests (Table 1). During this study, observations also revealed a high trace metals concentration in incense stored for a longer period (> 3 months to 2 years) compared to fresh or incense stored less than three months.

**Table 1:** ANOVA tests on the smoldered and non-smoldered non-perfumed and perfumed incense.

Source of variation	*SS*	*df*	*MS*	*F*	*P-value*	*F crit*
**A. Country-wise before and after smoldering non-perfumed and perfumed incense**						
Country-wise incense	118.94	31	3.83	6.74	< 0.01	1.51
metals	199.75	6	33.29	58.54	< 0.01	2.14
Error	105.77	186	0.56			
Total	424.46	223				
**B. Metals-wise, Governorate-wise before and after smoldering non-perfumed and perfumed incense**						
Metals-wise	391.18	25	15.64	23.07	< 0.01	1.59
Governorate-wise	20.46	5	4.09	6.03	< 0.01	2.28
Error	84.76	125	0.67			
Total	496.41	155				
**C. Oil-wise analysis before and after smoldering non-perfumed and perfumed incense**						
Smolder, non-smoldered incenses	11.44	3	3.81	8.79	0.0008	3.16
Metals, oil	24.97	6	4.16	9.59	0.0001	2.66
Error	7.81	18	0.43			
Total	44.22	27				
**D. Association of perfumed and non-perfumed smoldered incense to room size**						
Room sizes	1.52	6	0.253	8.119	0.011	4.28
Incenses (P, NP)	5.84	1	5.839	187.542	0.006	5.98
Error	0.18	6	0.031			
Total	7.54	13				

*SS* sum of squares, *df* degree of freedom, *MS* mean square, *F* calculated value, P-value for significance, *F crit* F critical table value, *P, NP* perfumed and non-perfumed incenses.

### Trace metals concentrations in PM_2.5_ of non-perfumed and perfumed smoldered incense

**Figure 3:**
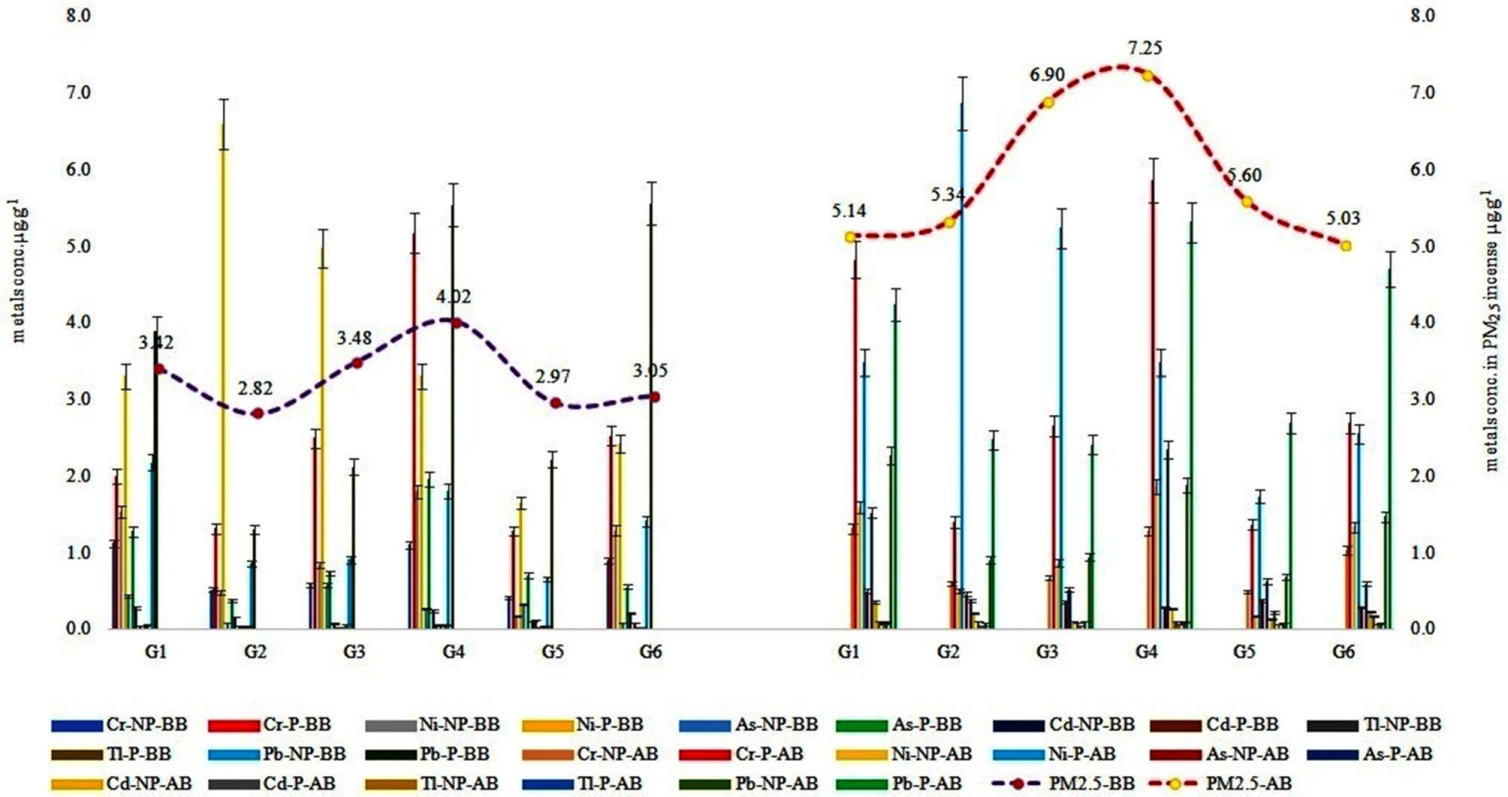
Trace metals and Governorate-wise before and after burning non-perfumed and perfumed incense. *G1–G6* Kuwait Governorates; *NP* non-perfumed incense, *P* perfumed incense; *BB* before burning; *AB* after burning.

This study further revealed the total mean trace metals concentrations (5.88 µg g^−1^) to be high in the PM_2.5_ dispersed smoldered incense in the indoor environment when compared to the total mean trace metals concentrations (3.29 µg g^−1^) in the PM_2.5_ of non-smoldered incense (Fig. 3). The Governorate-wise analysis revealed high trace metal concentrations in samples collected from Governorate-IV (G4) compared to other sites. Reasons for the high trace metals concentrations in PM_2.5_ and oil content in the incense collected from G4 site could be attributed to the resident’s constraints in living in such a populated area (Fig. 1) and small-sized housing that is influenced by external pollutants, improper ventilation and, quality of incense marketed in this area^20,24,27,30^. This was further validated by the results obtained from the indirect assessment that is dependent on variables that are discussed in this study. Statistical tests of ANOVA revealed significant differences between the country-wise, Governorate-metals-wise, and before and after smoldering in perfumed and non-perfumed incense (Table 1).

### Evaluation of respiratory ailments through indirect analysis of incense

**Figure 4:**
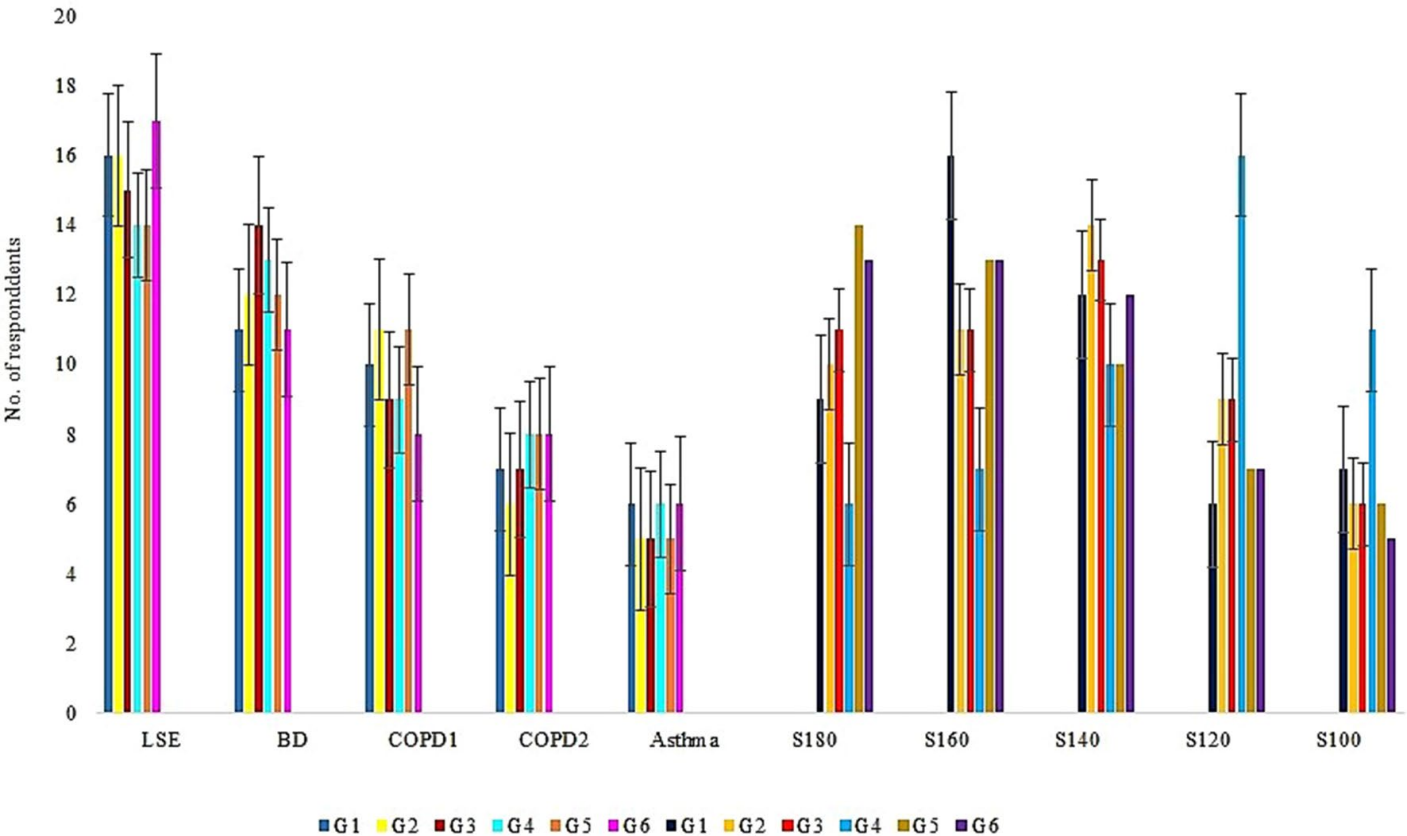
Number of respondents Vs respiratory ailments & room size. *LSE* low symptomatic effect; *BD* breathing difficulty, *COPD1* chronic obstructive pulmonary disease (cough); *COPD2* chronic obstructive pulmonary disease-(Emphysema), S100–S180: area of room in ft2.

The indirect analysis using a questionnaire on incense from the residents of Kuwait revealed responses ranging from mild to serious respiratory ailments. They are classified as (1) low symptomatic effect (LSE), (2) breathing difficulty (BD), (3) Chronic obstructive pulmonary disease COPD-1 (persistent cough), (4) COPD-2 (Emphysema), and (5) Asthma in Kuwait. The greatest number of cases was observed in LSE and the least with Asthma respondents. The largest numbers of people affected with respiratory difficulties were the residents exposed to smoldering incense inhabiting a room size of 140 ft^2^–160 ft^2^ for any given time (Fig. 4). Respondents (3–6 numbers) inhabiting a bigger sized room of 180 ft^2^ were observed with LSE than with asthmatic condition (Fig. S1). This indicated the diluted effect of incense smoke dispersed in a large-sized room used by the affluent and minimal number of residents compared to the concentrated effect of smoke in a small-sized room (100 ft^2^) inhabited by > 4 respondents without much ventilation. These findings validated the results of the pilot-scale experiment that standardized the effective inhalation of incense smoke dispersed within a distance between 0.5 ft and 1.0 ft length from the indoor inmates to the smoldering point. With adequate ventilation, it also quantified the time taken for the uniform smoke dispersion for 3 min, 7 min, 10 min, 12 min and 14 min from the non-perfumed and perfumed incenses in the sampled room area of 100 ft^2^, 120 ft^2^, 140 ft^2^, 160 ft^2^, 180 ft^2^ respectively. This uniform incense smoke dispersion was validated by the EPAM5000 monitor that measured the same PM_2.5_ nephelometric value at any point of the subjected room size over time (Fig. S2).

**Figure 5:**
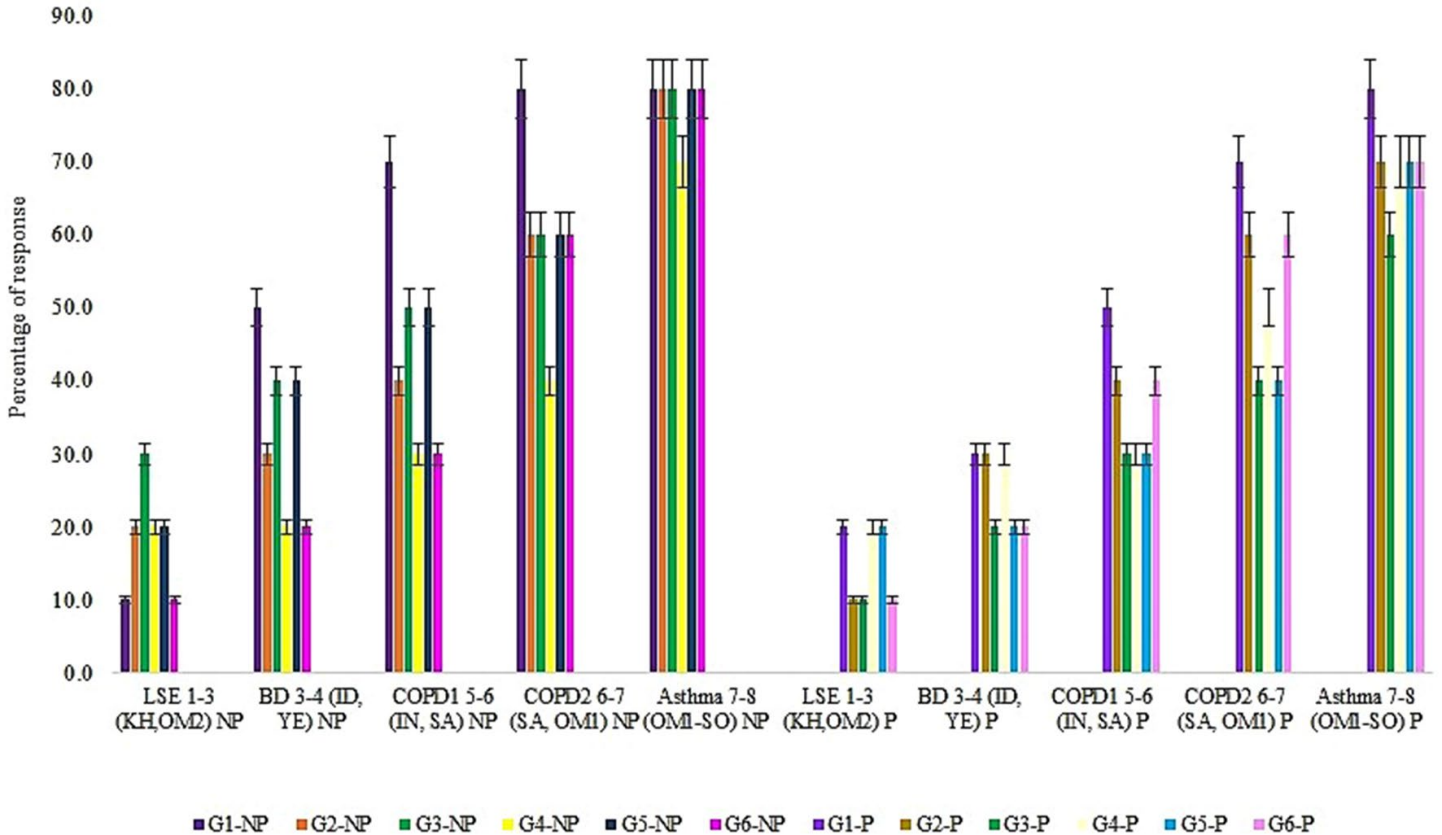
Governorate-wise type of incense causing respiratory ailments from non-perfumed and perfumed incense. *LSE* low symptomatic effect; *BD* breathing difficulty, *COPD1* chronic obstructive pulmonary disease (cough); *COPD2* chronic obstructive pulmonary disease-(emphysema); *G1–G6* Governorate areas.

The Governorate-wise analysis (Fig. 5) revealed a significant difference of low metals concentrations in G4 samples between the smoldering of incense and room size. This attributed to the influence from the outdoor environment, low sanitation, and dense population. Governorate-wise respondents was subjected to smoldered incenses in the small to large-sized rooms (100 ft^2^–180 ft^2^) equated to a range of metals concentrations and respiratory ailments namely, LSE (1–3: 0.26–1.47 µg g^−1^), BD (3–4: 0.38–1.57 µg g^−1^), COPD1 (5–6:0.65–1.82 µg g^−1^), COPD2 (6–7:0.97–2.49 µg g^−1^), Asthma (7–8:1.04–2.51 µg g^−1^). The numbers in brackets represented each group with the respective trace metals concentration ranges (from Fig. 2) and transformed to country-wise percentage distribution of the sampled perfumed and non-perfumed smoldered incenses (Fig. 5). Country-wise observations revealed the increasing sequence of the labeled group 1–3 (Frankincense of Cambodia, Oman-Dhofar, Indonesia), 3–4 (Frankincense of Indonesia, ‘Bakhour’ of Yemen), 5–6 (Frankincense of India and Saudi Arabia), 6–7 (Frankincense of Saudi Arabia and, Oman-Salalah), 7–8 (Frankincense of Oman-Salalah and, Bakhour of Somalia) (Fig. 5). A similar increasing sequence of respiratory ailments was noted in respondents subjected to smolder perfumed incense procured from countries such as Somalia, Oman, and Saudi Arabia. The incense from Yemen, Cambodia, and Oman-Dhofar revealed low variations in the respiratory ailments between LSE and BD in respondents (Fig. 5). The smoldered perfumed incense was found to elicit earlier respiratory ailments responses in respondents compared to the responses met with the smoldered non-perfumed incense (Fig. 5). Studies on the effect of organic constituents described by earlier investigators^6,11,22,28^ showed similar patterns. Interestingly, the trace metals concentrations were found high with respondents having Asthma- a mid-level respiratory ailment, when compared to the detrimental COPD1 and COPD2 (emphysema) ailments. Reasons to such high metals’ concentrations may be attributed to the pollen grains or mold absorbed heavy metals that cause Asthma^42^.

**Figure 6:**
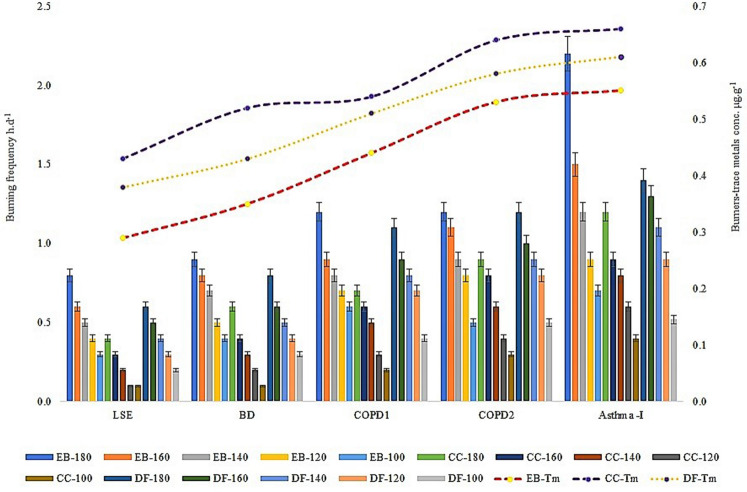
Effect of incense burners on room size, respiratory ailments, and trace metals concentrations. *LSE* low symptomatic effect; *BD* breathing difficulty, *COPD1* chronic obstructive pulmonary disease (cough); *COPD2* chronic obstructive pulmonary disease-(Emphysema), 100–180: area of room in ft2, *EB* electric burner, *CC* charcoal burner, *DF* direct flame, *Tm* trace metals.

Among the three main burners used, a significant difference between electric, coal, and direct flame that are used to smolder incense and ailments were observed. The use of a charcoal burner for burning incense in a room size ranging 100 ft^2^–180 ft^2^, with a burning frequency ranging 0.10–1.20 h day^−1^ continuously over a period of six months to one year was found adequate to cause respiratory ailments of LSE followed by BD, COPD1, COPD-2 and Asthma in the surveyed respondents. However, incense burning using the direct flame and electrical burner to produce such ailments in respondents inhabiting the same room size (100 ft^2^–180 ft^2^) required a longer burning frequency ranging 0.20–1.40 h day^−1^ and, 0.3–2.20 h day^−1^, respectively (Fig. 6). Thus, this novel study labels the smoke from incense using charcoal-burner as an additive hazardous part for the increased respiratory ailments compared to the use of lesser harmful electrical burners and direct flame. Additionally, 4% out of 300 respondents to questionnaire who used charcoal and electric burners in combinations to burn incense for more than 2–4 h day^−1^ in the indoor environment was affected with Emphysema (COPD2). This agreed with the earlier studies^1,10,30^ who observed people with respiratory illness after inhaling incense smoke over a continuous or longer exposure period compared to the lesser degree of ailments in occasional cigarette smokers. Furthermore, the incense burnt by a charcoal burner showed high trace metals concentrations (0.43–0.66 µg g^−1^) than with direct flame (0.38–0.61 µg g^−1^) and electric burners (0.29–0.59 µg g^−1^) (Fig. 6). Reasons that caused respiratory ailments from the charcoal-burner attributed to the release of carbon monoxide, trace metals, and other impurities, unlike the electric burner^14,34^. Furthermore, several residents (> 40% of the total respondents) revealed chronic ailments from breathing difficulties (BD) to Chronic Obstructive Pulmonary Disease (COPD-1) causing persistent cough due to the burning of incense directly by flame or using electric burners and exposed for > 6-8 h in the indoor environment. Studies^23,28,31^ observed such disorder of lung function associated with incense burning at home and air quality from the outdoor environment.

### Correlating the significance of direct and indirect (questionnaire) analysis of incenses

In an overall view, this study revealed significant synergism between the direct and indirect analysis in the following aspects: (1) the effect of increasing trace metals concentrations in the incenses (irrespective of smoldered or perfumed) was found proportionally higher with (a) the respondent’s health scale (Asthma > COPD2 > COPD1 > BD > LSE) (b) increasing quantity of incenses used per week (3–20 g/week), (c) incenses smoldered at increasing exposure (8 h day^−1^ to 24 h day^−1^), (d) increasing PM_2.5_ that were in line with the parameters such as the classified health scale, exposure period, quantity of incenses, smaller sized room, (e) the type of burners used to smolder the incenses (charcoal > direct flame > electric burner) and, (2) trace metals concentrations dispersed from the smoldered incenses was inversely proportional to spatial variations (i.e. increasing trace metals concentrations in smoldered incenses with decreasing room-size (120 ft^2^–180 ft^2^) (Fig. S3). Thus, this study prevents the use of low-quality incense and its continuous usage in a small dwelling area. It recommends lesser quantity and exposure period of incense used and preferably use electric burners usage over charcoal or direct flame to burn incense, despite their aromatic and beneficial traditional purposes.

## Conclusion

This study revealed the synergistic effects of (a) different types of incense from different countries of origin sold in the six Kuwait Governorate market, (b) variations before and after smoldering of non-perfumed and perfumed incense, (c) trace metals in PM_2.5_ dispersed smoke in small-large rooms (100 ft^2^–180 ft^2^) inhabited by residents, and (d) respiratory ailments (LSE, BD, COPD1, COPD2, Asthma). Trace metals concentrations exceeded the permissible limits in non-smoldered and smoldered incense except the frankincense from Dhofar-Oman (OM2). The present findings suggest to substantially reduce: (a) the use of perfumed-incense composed of a single or combination of oil at high concentrations, (b) the persistent exposure to perfumed incense during preparation and, while smoldering them, (c) the use of incense in a non-ventilated small-sized room (< 160 ft^2^) in a given period (< 10 min day^−1^), (d) the number of inmates (2–4 persons) for a given room size (100 ft^2^–180 ft^2^) when incense are smoldered, (e) the incense quantity burnt in a burner not exceeding 0.5–1.0 g day^−1^, (f) use of charcoal burner and replace by electric burner, (e) the incense from the excessive air-polluted country of origin, (f) inexhaustible use of incense in the rooms that is already subjected to indoor pollutants, (g) the use of incense on a long term basis and prevent respiratory ailment by the indoor residents.

## Supplementary Information

Supplementary File.

Supplementary File.

Supplementary File.

Supplementary File.
