# Supplementary figures and images for: Exposition of respiratory ailments from trace metals concentrations in incenses
Source: Sci Rep. 2021 May 21;11:10210. doi: 10.1038/s41598-021-89493-w (PMC8140077; doi:10.1038/s41598-021-89493-w)

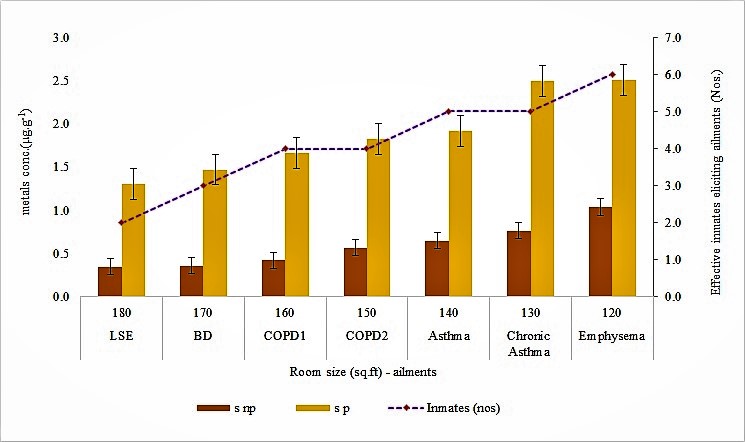

Supplement: Supplementary file 1 — Supplementary File. [file 41598_2021_89493_MOESM1_ESM.jpg]

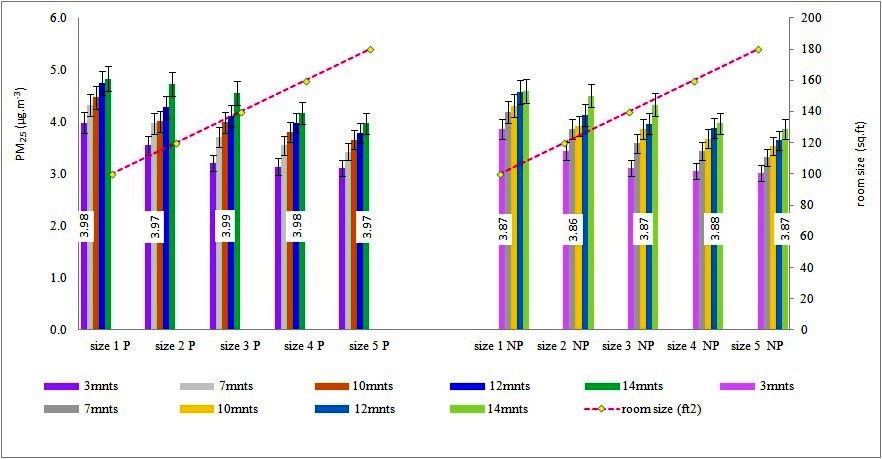

Supplement: Supplementary file 2 — Supplementary File. [file 41598_2021_89493_MOESM2_ESM.jpg]

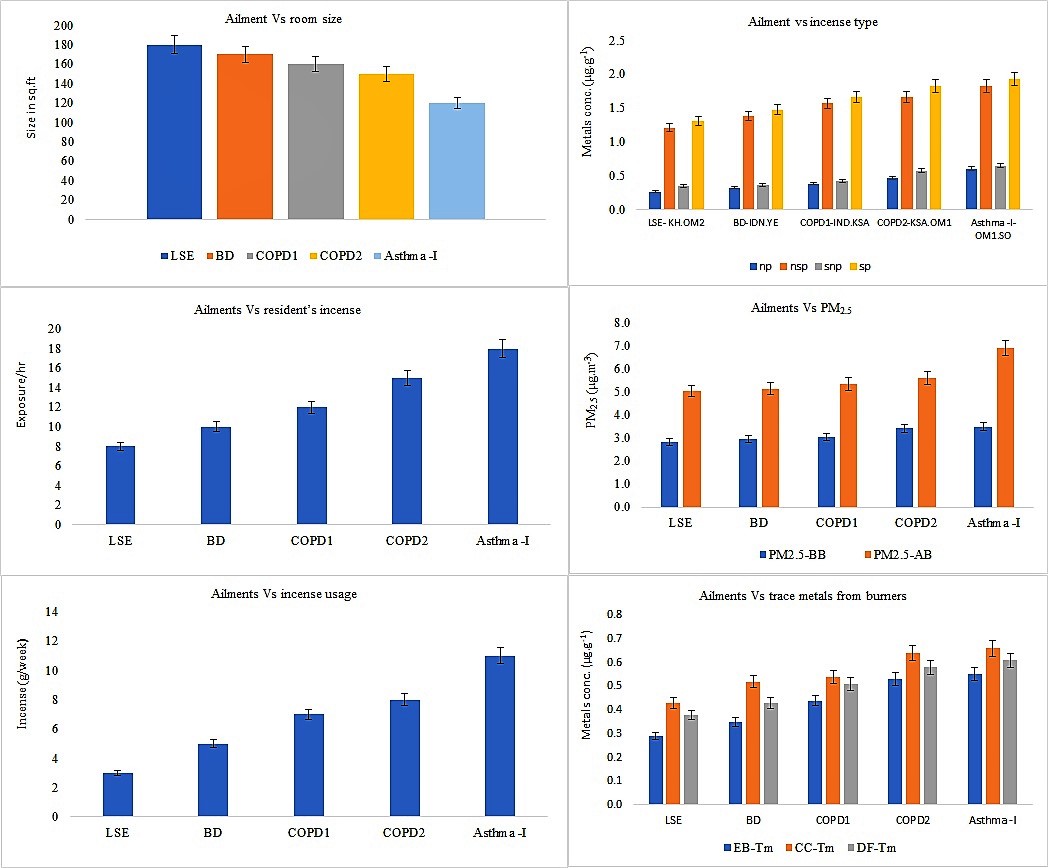

Supplement: Supplementary file 3 — Supplementary File. [file 41598_2021_89493_MOESM3_ESM.jpg]
