# Supplementary material for: Exposition of respiratory ailments from trace metals concentrations in incenses
Source: Sci Rep. 2021 May 21;11:10210. doi: 10.1038/s41598-021-89493-w (PMC8140077; doi:10.1038/s41598-021-89493-w)
Supplement: Supplementary file 4 — Supplementary File. [file 41598_2021_89493_MOESM4_ESM.docx]

### Exposition of respiratory ailments from trace metals concentrations in incenses

A.H. Bu-Olayan^1*^ and BV Thomas^1*^

**List of supplementary figures**

Fig. S1 Association between the trace metals concentrations in smoldered incense and room size

^LSE: low symptomatic effect; BD: breathing difficulty, COPD1: chronic obstructive pulmonary disease (cough); COPD2: chronic obstructive pulmonary disease-(Emphysema), 120-180: room size in sq.ft, Nos.: numbers (1-7 scales to corresponding metals concentrations of smoldered incenses)^

Fig. S2 Dispersion of PM_2.5_ from incense smoke in relation to room size and time

^P: perfume, NP: non-perfume, mnts: minutes, sq.ft: square feet, values in each group represent the PM2.5 against dispersion time and room sizes (sizes 1-5:100sq.ft-180 sq.ft)^

Fig. S3 Sliced data on the direct and indirect analysis of incenses

^LSE: low symptomatic effect, Breathing difficulty, COPD1-COPD2: chronic obstructive pulmonary disease, EB: electric burner, CC:charcoal burner, DF: direct flame, Tm: trace metals^
